# Caesarean Section

**DOI:** 10.1093/emph/eou031

**Published:** 2014-12-10

**Authors:** Wenda R. Trevathan, Karen R. Rosenberg

**Affiliations:** ^1^Department of Anthropology, New Mexico State University, Las Cruces, New Mexico USA; ^2^Department of Anthropology, University of Delaware, Newark, Delaware USA

## Rising caesarean section rates worldwide

Despite the World Health Organization’s (WHO) recommendation that caesarean section (c-section) rates should not exceed 15% [1], the high rates in some countries are cause for concern. For example, Italy, China, Mexico and Brazil all have rates higher than 36% [2] with great variation within each nation. The need for c-section has probably increased for many reasons, including rising rates of obesity, diabetes and maternal age, but rates more than twice the WHO recommendation probably reflect more than medical necessity. Although the lives of millions of mothers and infants have been saved by c-section, surgical delivery is not without costs. Risks to mothers include haemorrhage, pulmonary embolism, sepsis and death [3] as well as compromised breastfeeding and bonding [4]. C-sections may carry risk for infants regarding respiratory, metabolic, gastrointestinal and immune function [5]. Finally, there is increasing evidence for epigenetic changes with c-section [6] suggesting that it may not be just the mother and infant who are affected by surgical deliveries, but there may be transgenerational effects.

## Evolutionary perspectives on difficult birth

Many birth complications today can be attributed to modern lifestyles and technology, but challenging birth can be traced to the origin of bipedalism 5–7 million years ago [7]. Bipedalism restructured the pelvis, altering the birth mechanism so that the infant usually emerges occiput anterior, making it advantageous to have assistance [7]. Worldwide it is extremely unusual for women to give birth alone. Assistance at birth is deeply rooted in human evolutionary history and may explain evidence that social support has a positive impact on birth outcomes [8].

## Implications

Fear of birth or tocophobia is a common reason for electing to give birth by c-section. The evolutionary perspective argues that fear and the deeply rooted need for assistance during birth can often be alleviated with emotional support such as provided by doulas, thus avoiding unnecessary risky and costly c-section.
